# Low-dose alcohol exacerbates hyperdynamic circulation and shunting in non-alcoholic cirrhotic rats

**DOI:** 10.1042/BSR20240354

**Published:** 2024-07-19

**Authors:** Chon Kit Pun, Hui-Chun Huang, Ching-Chih Chang, Shao-Jung Hsu, Chiao-Lin Chuang, Yi-Hsiang Huang, Ming-Chih Hou, Fa-Yauh Lee

**Affiliations:** 1 School of Medicine, National Yang Ming Chiao Tung University, Taipei, Taiwan; 2 Division of Gastroenterology and Hepatology, Department of Medicine, Taipei Veterans General Hospital, Taipei, Taiwan; 3 Division of Holistic and Multidisciplinary Medicine, Department of Medicine, Taipei Veterans General Hospital, Taipei, Taiwan; 4 Division of General Medicine, Department of Medicine, Taipei Veterans General Hospital, Taipei, Taiwan

**Keywords:** alcohol, angiogenesis, liver cirrhosis, portal hypertension, portosystemic collaterals

## Abstract

Background: Portal hypertension affects hepatic, splanchnic and portosystemic collateral systems. Although alcohol is a well-known risk factor for liver cirrhosis, it also affects vascular contractility. However, the relevant effects on portal hypertension have not been evaluated in non-alcoholic cirrhosis. The present study aimed to investigate the impacts of low-dose alcohol on portal hypertension-related derangements in non-alcoholic cirrhotic rats.

Methods: Sprague-Dawley rats received bile duct ligation to induce cirrhosis or sham operation as controls. The chronic or acute effects of low-dose alcohol (2.4 g/kg/day, oral gavage, approximately 1.3 drinks/day in humans) were evaluated.

Results: The chronic administration of low-dose alcohol did not precipitate liver fibrosis in the sham or cirrhotic rats; however, it significantly increased splanchnic blood inflow (*P*=0.034) and portosystemic collaterals (*P*=0.001). Mesenteric angiogenesis and pro-angiogenic proteins were up-regulated in the alcohol-treated cirrhotic rats, and poorer collateral vasoresponsiveness to vasoconstrictors (*P*<0.001) was noted. Consistently, acute alcohol administration reduced splenorenal shunt resistance. Collateral vasoresponsiveness to vasoconstrictors also significantly decreased (*P*=0.003).

Conclusions: In non-alcoholic cirrhosis rats, a single dose of alcohol adversely affected portosystemic collateral vessels due to vasodilatation. Long-term alcohol use precipitated splanchnic hyperdynamic circulation, in which mesenteric angiogenesis played a role. Further studies are warranted to evaluate the benefits of avoiding low-dose alcohol consumption in patients with non-alcoholic cirrhosis.

## Introduction

Liver cirrhosis-related portal hypertension is a distinct form of hemodynamic dysregulation which develops along with the progression of liver fibrosis, although portal hypertension per se is not necessarily accompanied by liver fibrosis, as non-cirrhotic portal hypertension has also been identified. In addition to liver fibrosis, portal hypertension is characterized by increased hepatic resistance, hyperdynamic splanchnic blood inflow, abnormal vascular contractility, and pathologic angiogenesis. Hepatic fibrosis is a major pathologic component that compresses the intrahepatic vessels and hinders outflow of the portal system [1,2]. Pathological portosystemic collateral vessels thus gradually evolve to relieve the stagnant blood flow in the portal system. However, the shunting itself can cause lethal complications such as hepatic encephalopathy and gastroesophageal variceal bleeding. A recent study from the BAVENO VI-SPSS group demonstrated that large spontaneous portosystemic shunts were correlated with poor 1-year survival [3]. Consequently, the latest guidelines recommend noninvasive liver stiffness measurements to stratify clinically significant portal hypertension in daily practice, whereas hemodynamic measurements remain the gold standard to evaluate patients with chronic liver disease and portal hypertension [4].

Alcohol is a major risk factor for liver cirrhosis, and consumption of more than 40 g/day has been shown to increase the risk by up to 30% [5]. However, few studies have investigated the hemodynamic effects of alcohol on patients with non-alcoholic cirrhosis, and most of them have focused on liver fibrosis. The impact of light or moderate alcohol use on patients with pre-existing chronic liver disease is also unclear. For example, heavy alcohol use has been shown to accelerate the progression of liver cirrhosis in patients with chronic hepatitis C, while such an association was not seen in those with light or moderate alcohol use [6,7]. In addition, another study reported that mild to moderate alcohol consumption did not precipitate liver fibrosis in patients with chronic hepatitis B [8]. Due to the lack of sufficient evidence, recommendations can only be made based on the influence of alcohol on liver fibrosis and hepatocellular carcinoma [9–12]. Therefore, whether a light or moderate amount of alcohol consumption should be discontinued is also inconclusive. Alcohol affects vascular contractility and angiogenesis, both of which are pivotal pathophysiological factors in portal hypertension besides fibrogenesis [13,14]. Taken together, further investigations are needed to investigate the effects of a small amount of alcohol consumption on portal hypertension-related derangements.

Since low-dose alcohol per se may not precipitate liver fibrosis, a small amount of alcohol consumption in patients with non-alcoholic cirrhosis is not strictly prohibited by current guidelines [10,15]. Nevertheless, a small amount of alcohol may affect portal hypertension-related hemodynamic derangements through vascular mechanisms. This study thus evaluated the chronic or acute effects of low-dose alcohol, defined as the equivalent of 1.3 drinks per day in humans [15,16], on portal hypertension and relevant derangements in rats with common bile duct ligation (BDL)-induced chronic liver disease and liver cirrhosis.

## Methods

### Animal model: common BDL

Male Sprague-Dawley rats with a body weight (BW) ranging from 240 to 270 g at the time of surgery were used in the experiments. Common BDL was performed to induce biliary cirrhosis in the rats [1,2,17]. Under anesthesia (Zoletil 50 mg/kg BW, intramuscularly), the common bile duct was double ligated below the junction of the hepatic ducts and above the entrance of the pancreatic duct, respectively, and then cut between the ligatures. Liver cirrhosis developed four weeks after surgery [18]. The BDL rats received weekly vitamin K injections to avoid coagulation defects (50 μg/kg intramuscularly) [19].

This study was approved by Taipei Veterans General Hospital Animal Committee (IACUC 2016-008). All experimental procedures were performed at Taipei Veterans General Hospital Animal Laboratory and were conducted in accordance with the standard procedures indicated in the principles of laboratory animal care (Guide for the Care and Use of Laboratory Animals, DHEW publication No. (NIH) 85-23, rev. 985, Office of Science and Health Reports, DRR/NIH, Bethesda, MD, U.S.A.).

A total of 149 BDL rats and 11 sham-operated rats were used, all of which were anesthetized using Zoletil (50 mg/kg, intramuscularly). With regards to euthanasia, in the perfusion study the diaphragm was cut after all catheters had been inserted, and the rats died under anesthesia. In all other experiments, the rats died under anesthesia after blood collection at the end of the experiments.

### Experiment design

The effects of chronic low-dose alcohol administration on non-alcoholic cirrhosis were evaluated in the male Sprague-Dawley rats. Liver cirrhosis was induced by BDL, and sham-operated rats served as surgical controls. The rats were randomly allocated to receive either ethanol (2.4 g/kg/day, oral gavage) or vehicle (distilled water) from the 8th day after surgery, when liver fibrosis started to develop in the BDL group. The experiments were performed on the 35th day after surgery. Another series of studies was performed to evaluate the acute effects of low-dose alcohol. On the 35th day, after baseline hemodynamic measurements, the rats randomly received a single dose of ethanol (2.4 g/kg, oral gavage) or vehicle (distilled water). One hour later, the parameters were measured again and the effects of acute alcohol administration were analyzed.

The dose of alcohol was determined based on a previous study on humans and rats, which showed that the blood concentration of alcohol was approximately 46.7 mg/dl at 30 min after the oral administration of 2.4 g/kg of ethanol in rats [15]. A 60 kg person would have the same blood alcohol concentration 30 min after consuming 17.6 g of alcohol, which is equivalent to 1.3 drinks as defined by the National Institute on Alcohol Abuse and Alcoholism (NIAAA). In patients with chronic hepatitis C, daily alcohol intake of 1–20 g/day is considered to be minimal [16]. In addition, this is considered to be ‘safe’ drinking, since the upper limit is 2 drinks per day for people [20].

### Measurement of systemic and portal hemodynamics

The methods used to measure systemic and portal hemodynamics were adopted from previous studies in our lab [1,2]. Continuous recordings of mean arterial pressure (MAP) and heart rate were performed on a multi-channel recorder (MP45, Biopac Systems Inc., Goleta, CA, U.S.A.) with a PE-50 catheter cannulated into the right carotid artery. The external zero reference was placed at the level of the mid-portion of the rat. An 18-gauge catheter was cannulated into the mesenteric vein to measure portal pressure.

A pulsed-Doppler flow transducer (T206 Small Animal Blood Flow Meter, Transonic Systems Inc., Ithaca, NY, U.S.A.) was used to measure flow in the superior mesenteric artery (SMA) [1]. An adequately sized transducer was placed over the portal vein as proximal to the liver as possible to measure portal flow.

Cardiac output (CO) was measured by thermodilution, as previously described [1,2,21]. Cardiac index (CI, ml/min/100 g BW) was calculated as CO per 100 g BW. Systemic vascular resistance (mmHg/ml/min/100 g BW) was calculated by dividing MAP by CI. SMA resistance (mmHg/ml/min/100 g BW) was calculated as (MAP-portal pressure)/SMA flow per 100 g BW. Portal resistance (mmHg/ml/min/100g BW) was calculated as portal pressure/portal flow per 100 g BW.

### Hepatic fibrosis determination with Sirius red staining

Liver paraffin sections were stained using a Sirius red staining kit (Polysciences Inc., Warrington, PA, U.S.A.). ImageJ software (available for download from the National Institutes of Health (http://rsb.info.nih.gov/ij/)) was used to measure the percentage of Sirius red-stained areas, as previously described [1,2].

### Mesenteric and hepatic hematoxylin and eosin staining

Mesenteries and livers were fixed in 10% formalin, embedded in paraffin, sectioned at 5 μm, and stained with hematoxylin and eosin (H&E).

### Immunofluorescent study for mesenteric vascular density

Mesenteric angiogenesis was quantified by CD31-labelled microvascular networks in rat mesenteric connective tissue windows according to previous studies [1,2]. From each rat, at least four mesenteric windows (wedge-shaped regions of connective tissue bordered by the intestinal wall and ileal blood vessel pairs) were dissected free, washed in phosphate-buffered saline, dried on gelatin slides, and fixed in 100% MeOH (−20°C for 30 min). The slides were then incubated overnight at 4°C with the primary antibody mouse anti-rat CD31-biotin (AbD Serotec, Oxford, U.K.). A secondary antibody (CY2-conjugated streptavidin; Jackson ImmunoResearch, West Grove, PA, U.S.A.) was then applied for 1 hour at room temperature. Whole membrane images were obtained and thresholded using ImageJ software.

### Color microsphere method for portosystemic shunting degree analysis

Portosystemic shunting degree was determined using the technique described by Chojkier and Groszmann [22] and substituting color for radioactive microspheres [23]. Under anesthesia, 30,000 of 15-μm yellow microspheres (Dye Track; Triton Technology, San Diego, CA, U.S.A.) were slowly injected into the spleen. The rats were euthanized, and the livers, lungs, and spleens were dissected. The number of microspheres in each tissue sample was calculated according to the manufacturer. Shunting severity was calculated as lung microspheres/(liver microspheres plus lung microspheres).

### Western analysis

The mesentery, which is mainly composed of blood vessels and connective tissue, was isolated immediately after hemodynamic measurements, and cut between soft tissue, small intestine and superior mesenteric vein (SMV). The isolated mesentery was immediately frozen in liquid nitrogen and stored at −80°C until required. The primary antibodies used in this study are shown in Supplementary Figure.

### In situ perfusion preparation

The *in situ* mesenteric perfusion technique was modified from the *in vitro* SMA perfusion technique described previously [24,25]. In short, the SMA was perfused with an 18-gauge Teflon cannula. The perfusate flowed out of the splanchnic system via a 16-gauge Teflon cannula at the proximal end of the SMV. A ligature was tied over the SMV as close to the portal vein as possible to exclude the liver and collateral vessels from perfusion. The perfusion was performed in a warm chamber (37 ± 0.5°), and the temperature was monitored. The rats then received *in situ* perfusion with acetylcholine (10^−10^, 10^−9^, 3 × 10^−9^, 10^−8^, 3 × 10^−8^, 10^−7^ M) with phenylephrine (10 μM) pre-contraction or arginine vasopressin (AVP) (10^−10^, 10^−9^, 3 × 10^−9^, 10^−8^, 3 × 10^−8^, 10^−7^ M). The dose response curves of each rat were recorded.

*In situ* liver perfusion was performed as previously described with minor modifications [24,26]. The liver was perfused via a 16-gauge Teflon cannula over the portal vein. The perfusate flowed out from two 16-gauge Teflon cannulas at both jugular veins. Pneumothorax was created to prevent lung perfusion.

### Portosystemic collateral vascular bed perfusion

*In situ* portosystemic collateral perfusion was performed as previously described [27,28]. An 18-gauge Teflon cannula was inserted into the distal SMV, and two 16-gauge cannulas were placed over both jugular veins. A second loose ligature around the portal vein was tied to exclude liver perfusion.

### Drugs

Ethanol, endothelin-1 (ET-1) and AVP were purchased from Merck KGaA (Darmstadt, Germany). All solutions were freshly prepared on the days of the experiment.

### Statistical analysis

All results are expressed as mean ± SEM. Changes in perfusion pressure (mmHg) over baseline were calculated for each concentration in each preparation. Statistical analyses were performed using an unpaired Student’s *t*-test as appropriate. SPSS version 21 software for Windows (SPSS Inc., Chicago, IL, U.S.A.) was used for analyses. Results were considered statistically significant at a two-tailed *P*-value of less than 0.05.

## Results

### Chronic administration of low-dose alcohol

**Figure 1:**
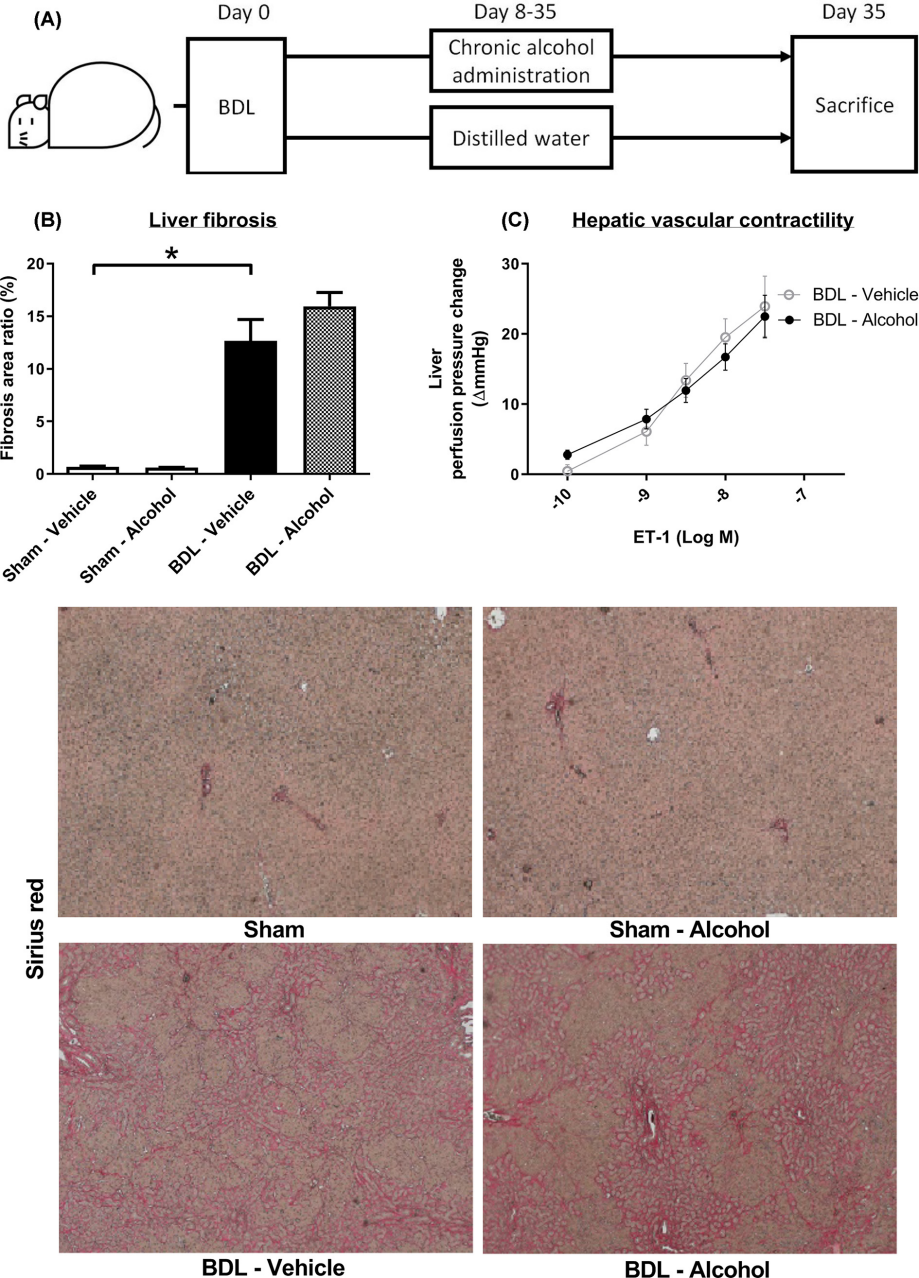
Effects of chronic alcohol administration on the liver (**A**) BDL and sham rats were randomly allocated to receive ethanol or vehicle (distilled water) from the 8th day after surgery. (**B**) The severity of liver fibrosis was evaluated by fibrosis area ratio. The area ratio was significantly increased in the BDL-vehicle group compared with the sham-vehicle group. In the chronic alcohol-treated group, the area ratio was not affected compared with the BDL-vehicle group (*n* = 6, 7). (**C**) The hepatic perfusion pressure was tested *in situ* with ET-1 infusion. The vascular responsiveness to ET-1 was not significantly different between the BDL-vehicle and BDL-alcohol groups (*n* = 4, 5).

Rats received BDL to induce liver cirrhosis and portal hypertension, with sham-operated rats as surgical controls. The rats were further randomly allocated to receive ethanol or vehicle (distilled water) per day from the 8th day after surgery, when liver fibrosis had developed in the BDL rats. The results were evaluated on the 35th day after surgery (Figure 1A).

Table 1 depicts the results of BW, systemic and portal hemodynamic parameters of the experimental groups. The cirrhotic rats had a significantly lower BW compared with the sham-operated rats. In addition, MAP and systemic vascular resistance decreased while CI increased significantly in the cirrhotic groups, reflecting the features of hyperdynamic circulation in portal hypertension. A small amount of alcohol administration did not affect portal pressure, MAP or heart rate in the sham or BDL groups. However, systemic vascular resistance decreased and CI increased significantly in the alcohol-treated cirrhotic rats. These results suggested that low-dose alcohol aggravated systemic vasodilatation in the cirrhotic rats.

**Table 1:** Body weight, hemodynamic parameters and plasma biochemistry parameters in Sham or bile duct ligation (BDL) rats treated with vehicle (distilled water) or alcohol

	Sham - Vehicle	Sham - Alcohol	BDL - Vehicle	BDL - Alcohol	*P* value^*^
	*n*=6	*n*=5	*n*=7	*n*=7	
**BW (g)**	484 ± 12	430 ± 7	358 ± 15^†^	357 ± 19	0.963
**MAP (mmHg)**	107 ± 3	110 ± 4	85 ± 8^†^	82 ± 7	0.774
**HR (beats/min)**	252 ± 12	252 ± 10	243 ± 6	258 ± 9	0.197
**PP (mmHg)**	6.8 ± 0.5	6.7 ± 1.1	13.3 ± 1.4^†^	13.1 ± 1.4	0.926
**Systemic circulation**					
CI (ml/min/100 g)	28.1 ± 1.1	28.5 ± 1.7	41.3 ± 2.6^†^	54.4 ± 2.1^*^	0.002
SVR (mmHg/ml/min/100 g)	3.8 ± 0.2	3.9 ± 0.2	2.1 ± 0.2^†^	1.5 ± 0.2^*^	0.04
**Splanchnic system**					
SMA flow (ml/min/100 g)	3.8 ± 0.2	5.0 ± 0.2	5.4 ± 0.9	7.8 ± 0.5^*^	0.034
SMA resistance (mmHg/ml^/^min/100 g)	26.9 ± 1.9	20.6 ± 1.0	16.0 ± 3.8^†^	9.3 ± 1.0	0.093
**Hepatic system**					
Hepatic inflow (portal site) (ml/min/100 g)	6.6 ± 1.9	3.5 ± 0.5	6.8 ± 2.0	7.9 ± 0.6	0.532
Hepatic vascular resistance (mmHg/ml^/^min/100 g)	1.1 ± 0.1	2.0 ± 0.4	2.6 ± 0.9	1.7 ± 0.1	0.263
**Plasma biochemistry parameters**					
AST (U/L)	109 ± 8	145 ± 24	509 ± 30^†^	668 ± 146	0.312
ALT (U/L)	58 ± 3	67 ± 7	100 ± 10^†^	130 ± 27	0.335
Total bilirubin (mg/dl)	0.2 ± 0	0.64 ± 0.37	8.3 ± 1.9^†^	8.4 ± 1.2	0.946

Abbreviations: BDL, bile duct ligation; BW, body weight; CI, cardiac index; DW, distilled water (control); HR, heart rate; MAP, mean arterial pressure; PP, portal pressure; SMA, superior mesenteric artery; SVR, systemic vascular resistance.

* Alcohol-treated group compared with parallel control group (BDL-vehicle).

† *P*<0.05, BDL-vehicle compared with sham-vehicle group.

### Effects of low dose alcohol on hepatic system

Hepatic vascular resistance and hepatic blood flow (portal side) were not affected by a small amount of alcohol (Table 1). Hepatic resistance is determined by both structural and functional components, and both components were then evaluated. The main structural factor was liver fibrosis, which was assessed by fibrosis area ratio (Figure 1B). The area ratio was significantly increased in the BDL-vehicle group compared with the sham-vehicle group (0.68 ± 0.08% vs 12.7 ± 2.03%, *P*<0.001). The severity of liver fibrosis was not affected in the chronic alcohol-treated group (12.7 ± 2.0% vs 15.9 ± 1.3%; *P*=0.192).

The liver biochemistry parameters of the experimental groups are shown in Table 1. The ALT, AST, and total bilirubin levels were significantly increased in the BDL groups. However, there were no significant differences between the alcohol- and DW-treated groups in liver biochemistry parameters.

Functional component abnormalities are caused by overt vasoconstriction in the liver. Hepatic vascular contractility was evaluated by *in situ* liver perfusion (Figure 1C), which showed no significant difference in vascular responsiveness to ET-1 between the two groups (*P*=0.909). Taken together, low-dose alcohol administration did not induce observable liver injury.

### Effects of low-dose alcohol on the mesenteric system

In liver cirrhosis, splanchnic inflow increases and resistance decreases due to abnormal angiogenesis (structural component) and vasodilatation (functional component). Splanchnic hyperdynamic circulation is a feature in portal hypertension. Interestingly, alcohol further exacerbated splanchnic hyperdynamic circulation in the cirrhotic rats in the present study (Table 1).

**Figure 2:**
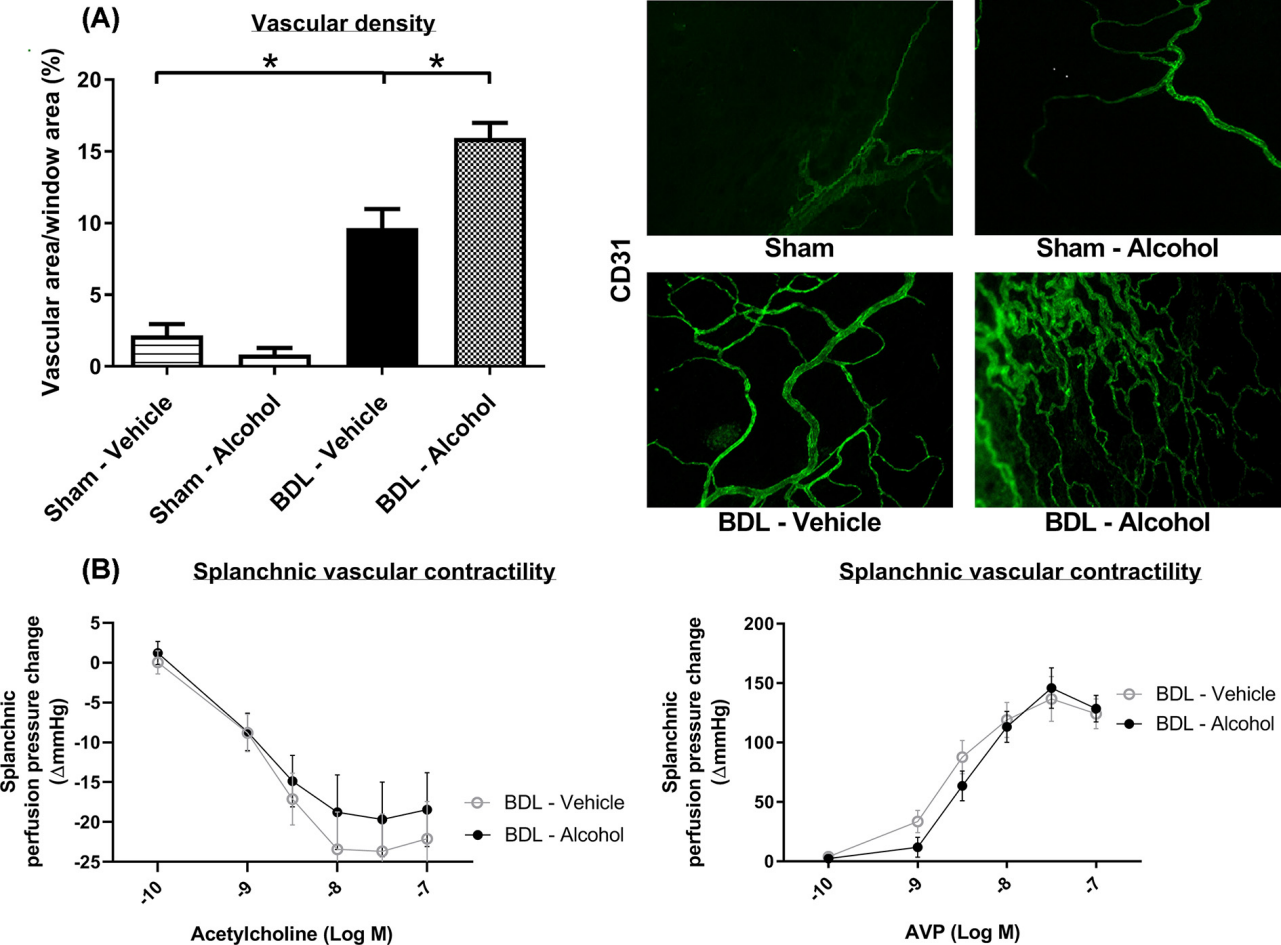
Effects of chronic alcohol administration on the mesentery (**A**) Mesenteric angiogenesis was evaluated by the vascular density of mesenteric windows. In the cirrhotic rats, the mesenteric vascular density was markedly higher than in the sham rats. The vascular area per unit area of mesenteric window was significant higher in the chronic alcohol-treated BDL group (*n* = 6, 6, 5, 6; Sham-vehicle; Sham-alcohol; BV, BDL-vehicle; BA, BDL-alcohol). (**B**) The splanchnic perfusion pressure was tested *in situ* with acetylcholine and AVP infusion. The vascular responsiveness to acetylcholine and AVP was not significantly different between the BDL-vehicle and BDL-alcohol groups (*n* = 5, 5).

To investigate the effects of alcohol on splanchnic hemodynamics, mesenteric angiogenesis was evaluated by the vascular density of mesenteric windows (Figure 2A). In the cirrhotic rats, the mesenteric vascular density increased significantly (sham-vehicle vs BDL-vehicle: 3.2 ± 1.2% vs 9.6 ± 1.3%, *P*=0.006). Chronic alcohol administration further enhanced mesenteric angiogenesis in the cirrhotic rats (BDL-vehicle vs BDL-alcohol: 9.6 ± 1.3% vs. 15.9 ± 1.1%; *P*=0.006).

Splanchnic vascular contractility was determined using *in situ* splanchnic perfusion via acetylcholine and AVP infusion (Figure 2B). There were no significant differences in vascular responsiveness to acetylcholine (*P*=0.544) and AVP (*P*=0.281) between the BDL-vehicle and BDL-alcohol groups.

**Figure 3:**
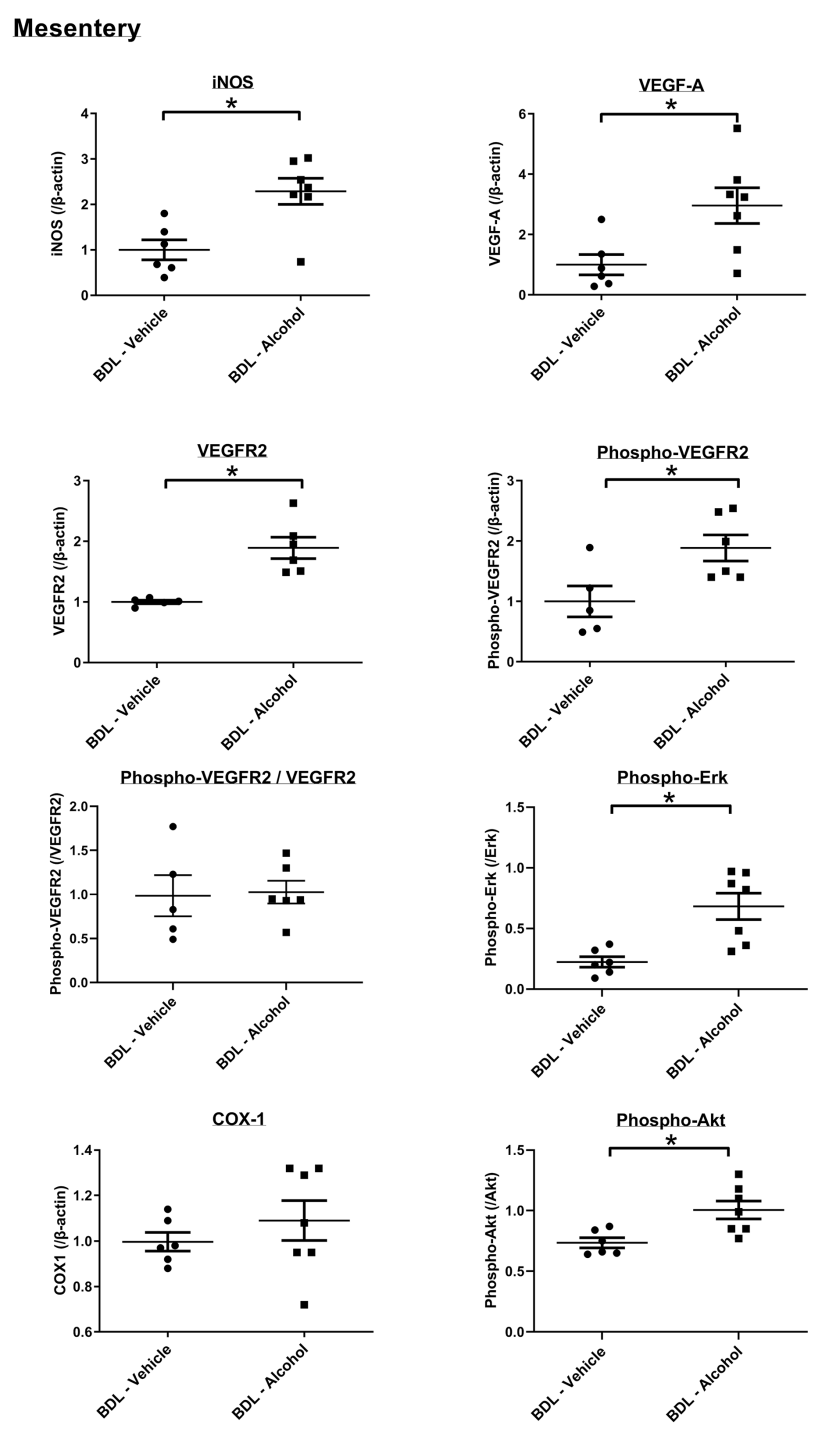
The angiogenic protein expressions in the mesentery of the BDL rats Chronic alcohol administration up-regulated VEGF-A, VEGFR2 (*n* = 5, 6), phospho-VEGFR2 (*n* = 5, 6), phospho-Akt, phospho-Erk and iNOS expressions. COX1 expressions were not significantly different (*n* = 6, 7).

Mesenteric protein expressions are shown in Figure 3, and whole blots are shown in Supplementary Figure. The angiogenesis-related factors vascular endothelial growth factor (VEGF)-A, VEGFR2, phospho-VEGFR2, phospho-Akt, phospho-Erk and iNOS were significantly up-regulated in the alcohol-administered group (BDL-vehicle vs BDL-alcohol (/β-actin), VEGF-A: 1.00 ± 0.34 vs 2.96 ± 0.59, *P*=0.02; VEGFR2: 1.00 ± 0.03 vs 1.89 ± 0.18, *P*=0.001; phospho-VEGFR2: 1.00 ± 0.26 vs 1.88 ± 0.22, *P*=0.026; phospho-Akt (/Akt): 0.74 ± 0.04 vs 1.01 ± 0.07, *P*=0.01; phospho-Erk (/Erk): 0.22 ± 0.04 vs 0.68 ± 0.11, *P*=0.004; iNOS: 1.00 ± 0.22 vs 2.29 ± 0.29, *P*=0.005). However, there was no significant difference in phospho-VEGFR2/VEGFR2 between the two groups (BDL-vehicle vs BDL-alcohol: 0.98 ± 0.23 vs 1.03 ± 0.13, *P*=0.871). In addition, the protein expression of COX1 was not significantly different between the two groups (COX-1: 1.00 ± 0.04 vs 1.09 ± 0.09, *P*=0.394). In addition, there were no significant differences in VEGF-C, VEGFR3, and phospho-eNOS expressions between the two groups (Supplementary Figure S1).

### Effects of low-dose alcohol on the collateral system

**Figure 4:**
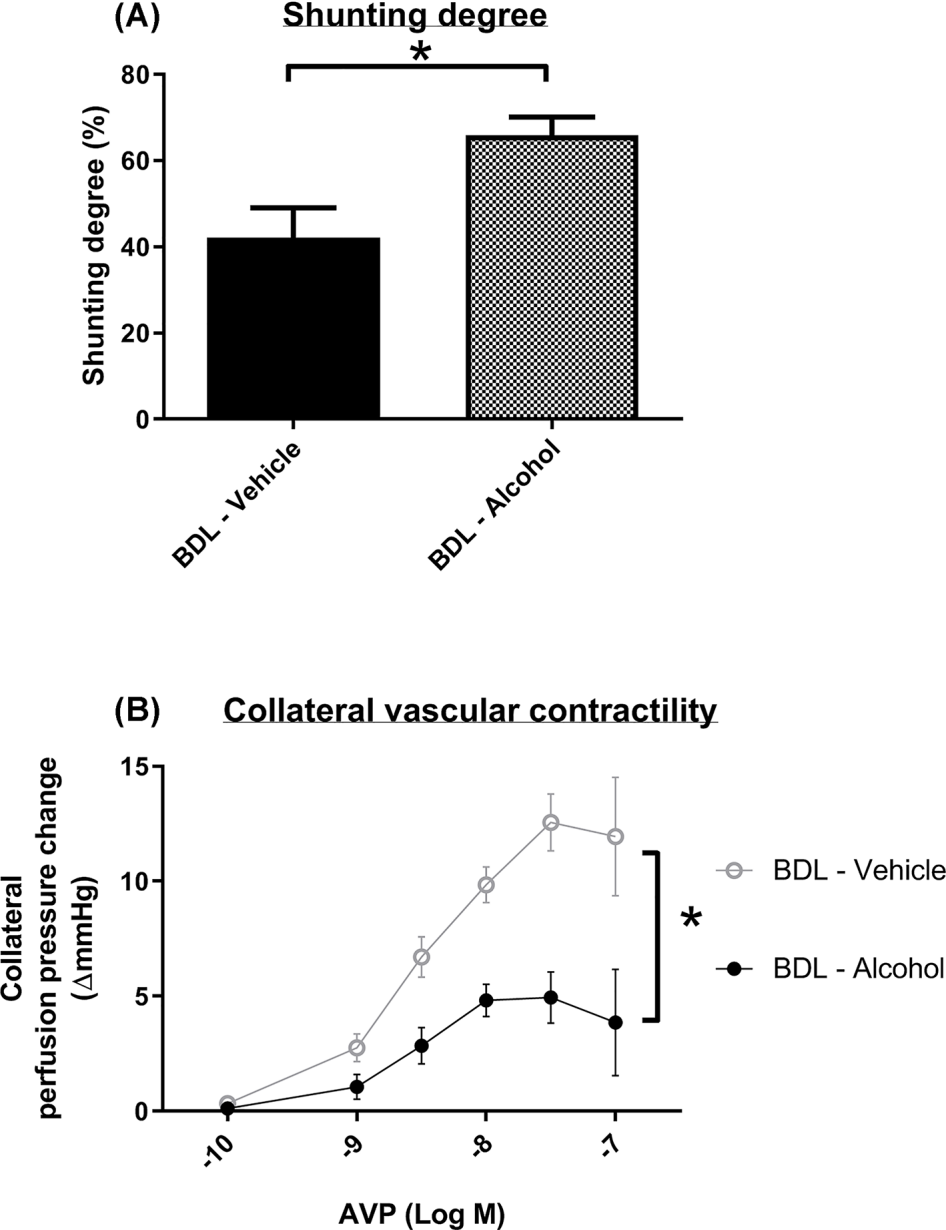
Effects of chronic alcohol administration on portal-systemic collateral shunting and vascular contractility (**A**) The degree of shunting was evaluated using the color microsphere method, which showed that the degree of shunting was significantly increased in the chronic alcohol-treated group (*n* = 6, 7). (**B**) The collateral perfusion pressure was tested *in situ* with AVP infusion. The vascular responsiveness to AVP significantly decreased in the chronic alcohol-treated BDL rats (*n* = 4, 5).

Portosystemic collateral shunting vessels are associated with variceal bleeding and hepatic encephalopathy. Shunting severity was evaluated using the color microsphere method (Figure 4A). Collateral shunting degree increased significantly in the chronic alcohol-treated group (42.1 ± 6.9% vs 65.8 ± 4.3%, *P*=0.012). Vascular contractility was evaluated using *in situ* collateral perfusion, which showed that vascular responsiveness to AVP significantly decreased in the chronic alcohol-treated group (*P*<0.001) (Figure 4B). These results suggested that a small amount of alcohol worsened collateral shunting through a vasodilatation effect.

### Acute administration of low-dose alcohol in rats with BDL-induced liver cirrhosis

**Figure 5:**
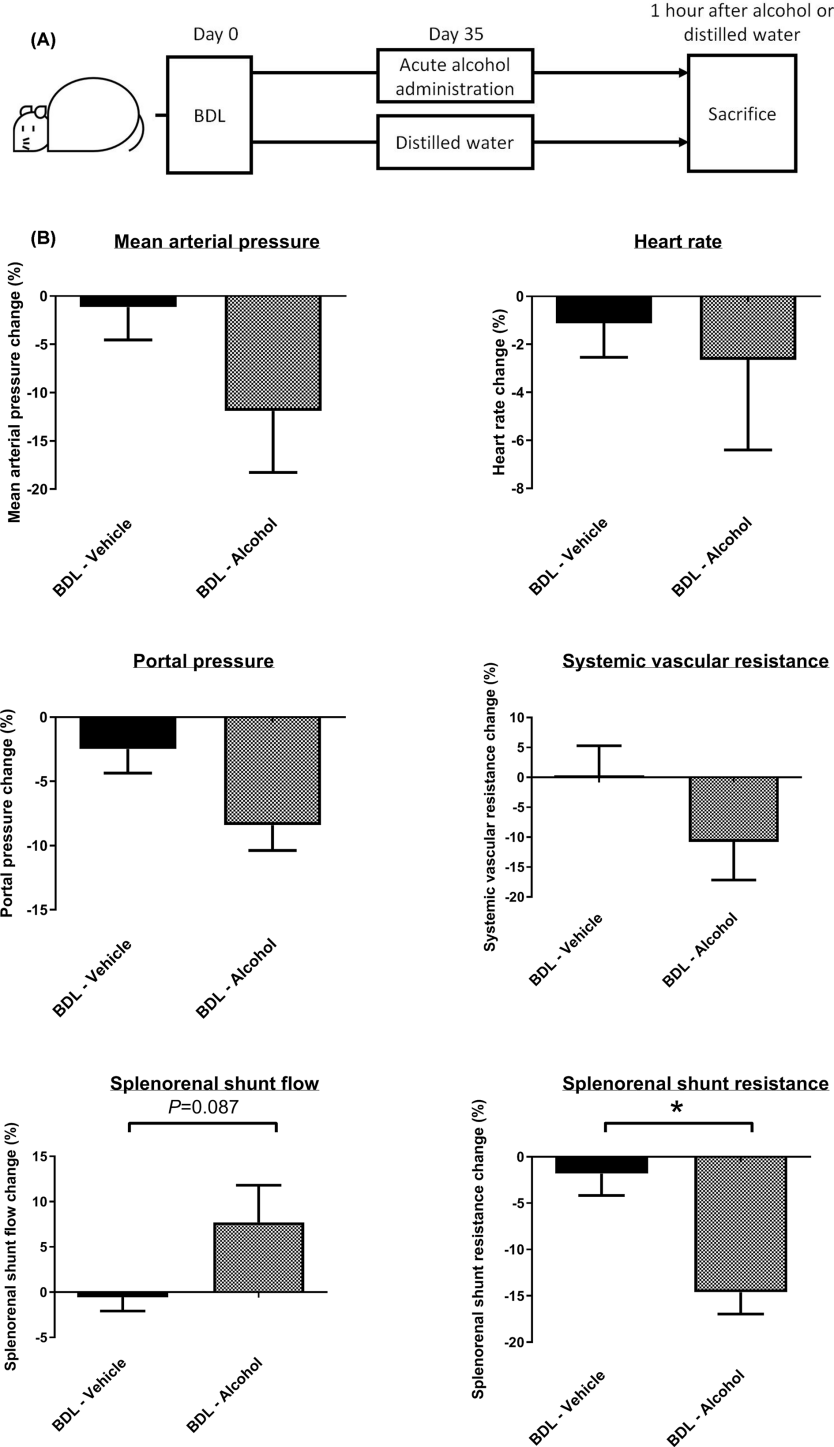
Effects of acute alcohol administration on hemodynamic parameters in the BDL rats (**A**) The BDL rats were randomly allocated to receive a single dose of alcohol or vehicle (distilled water) on the 35th day after BDL. (**B**) Mean arterial pressure, portal pressure and systemic vascular resistance tended to decrease. Splenorenal shunt flow tended to increase, and splenorenal shunt resistance decreased significantly (*n* = 6, 6).

The BDL rats randomly received a single dose of ethanol or vehicle (distilled water) on the 35th day after surgery, at which time significant liver cirrhosis and portal hypertension had developed. The results were evaluated 1 h after the administration of ethanol or vehicle (Figure 5A). Alcohol was administered in the cirrhotic rats with the same dosage and route as in those receiving chronic treatment. The impact of acute alcohol administration on the hemodynamic parameters is shown in Figure 5B. One hour after alcohol administration, there was a trend toward a reduction in MAP (BDL-vehicle vs BDL-alcohol, MAP change (%): -1.14 ± 3.40 vs -11.9 ± 6.40, *P*=0.167), which was consistent with the findings of chronic treatment that alcohol induced peripheral vasodilatation.

It is difficult to assess the effects of acute alcohol administration on collateral shunting using the microsphere method. Therefore, we evaluated the flow and resistance of splenorenal shunt (SRS) instead, the most prominent collateral vessel in rodents. The results showed that SRS resistance decreased significantly (BDL-vehicle vs BDL-alcohol, SRS resistance change (%): -1.810 ± 2.37 vs -14.6 ± 2.34, *P*=0.003). In addition, a trend of an increase in SRS flow was observed (BDL-vehicle vs BDL-alcohol, SRS flow change (%): -0.57 ± 1.52 vs 7.71 ± 4.10, *P*=0.087). The consistent data in the acute and chronic studies suggested that a small amount of alcohol administration exacerbated portosystemic collateral shunting.

**Figure 6:**
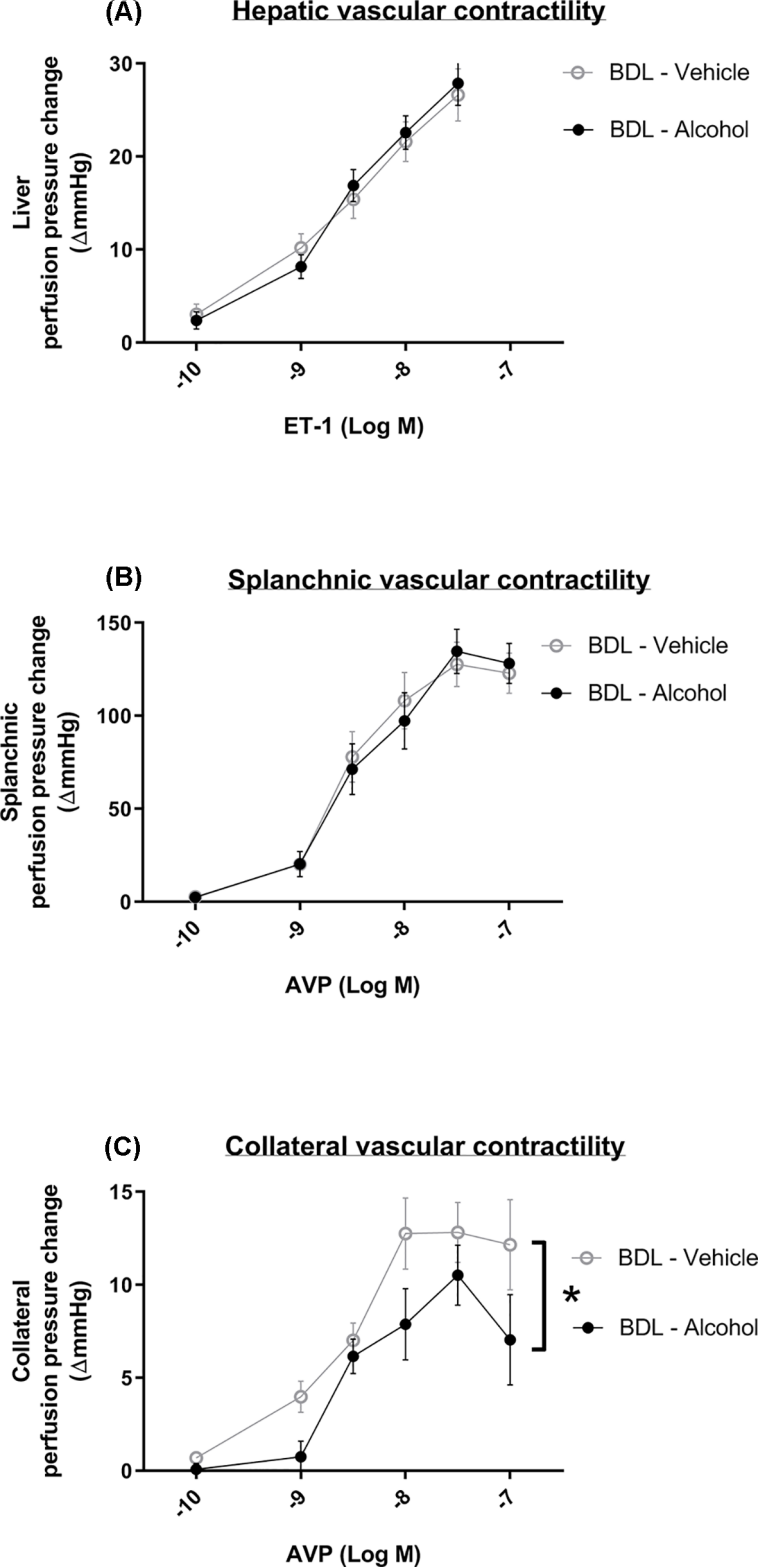
Effects of acute alcohol administration on hepatic, splanchnic, and collateral vascular contractility in the BDL rats (**A**) The hepatic perfusion pressure was tested *in situ* with ET-1 infusion. The vascular responsiveness to ET-1 was not significantly different between the BDL-vehicle and BDL-alcohol groups (*n* = 5, 7). (**B**) The splanchnic perfusion pressure was tested *in situ* with AVP infusion. The vascular responsiveness to AVP was not significantly different between the BDL-vehicle and BDL-alcohol groups (*n* = 5, 5). (**C**) The collateral perfusion pressure was tested *in situ* with AVP infusion. The vascular responsiveness to AVP significantly decreased in the acute alcohol-treated group (*n* = 5, 5).

The direct effects of acute alcohol administration on different vascular territories were also evaluated. The results showed that there were no significant differences in hepatic or splanchnic vascular responsiveness between the BDL-vehicle and BDL-alcohol groups (Figure 6A,B). Interestingly, vascular responsiveness to AVP significantly decreased in the collateral system (*P*=0.003). In summary, the acute administration of low-dose alcohol aggravated collaterals through collateral vasodilatation.

## Discussion

In the present study, a small amount of alcohol induced collateral and systemic vasodilatation and worsened portosystemic collateral shunting. Moreover, long-term ingestion of low-dose alcohol exacerbated splanchnic hyperdynamic circulation via extrahepatic pathological angiogenesis. Nevertheless, low-dose alcohol did not worsen the progression of liver fibrosis. Increasing splanchnic inflow and collateral vasodilatation have detrimental effects in cirrhotic patients. For example, increased flow in gastroesophageal varices can lead to varices rupture. This implies that the adverse effects of alcohol in cirrhotic patients may be underestimated if only the severity of liver fibrosis is monitored.

Splanchnic hyperdynamic circulation is an important component of portal hypertension. Abnormal splanchnic blood inflow can be caused by structural (angiogenesis) and functional (vasodilation) components. During the development of portal hypertension, pathological angiogenesis occurs over the splanchnic area. This initially aims to divert the stagnant splanchnic blood flow, but in the long run drains more blood flow into the portal system. Several factors are involved in the process of neovascularization, of which VEGF plays a pivotal role [29]. In primary human microvascular endothelial cells, alcohol has been shown to significantly increase VEGF mRNA expression [30]. In addition, alcohol has been shown to promote neovascularization via a VEGF pathway in swine hearts [31], and also to stimulate VEGF expression and angiogenesis in human fibrosarcoma cells [13]. A previous study demonstrated that moderate alcohol intake could induce angiogenesis by up-regulating VEGF in chick chorioallantoic membranes [32]. Moreover, another study reported that long-term alcohol exposure could potentiate neovascularization in rat livers by modulating the VEGF pathway [33]. A human study also reported that individuals with alcoholism had elevated serum VEGF concentrations [34]. It is worth noting that alcohol affects the VEGFR2 pathway via multiple mechanisms. One study demonstrated that long-term alcohol consumption for 36 weeks induced VEGFR2 up-regulation but not phosphorylation in rats [33]. In our study, chronic alcohol intake resulted in the concurrent up-regulation of VEGFR2 and phospho-VEGFR2, which is consistent with the previous findings. This result implies that long-term alcohol use up-regulates VEGFR2 and subsequently phospho-VEGFR2 expression in the mesentery instead of merely promoting phosphorylation of VEGFR2. Furthermore, alcohol significantly increased mesenteric vascular density. Taken together, these results suggest that alcohol worsens splanchnic hyperdynamic circulation via mesenteric angiogenesis and may be associated with up-regulation of the VEGF signaling pathway.

We also found that chronic alcohol administration up-regulated iNOS in the mesentery of the cirrhotic rats. The interaction of alcohol and NO is complex. In bovine aortic endothelial cells and human umbilical endothelial cells, ethanol has been shown to increase NO production through the up-regulation of eNOS [35]. On the other hand, short-term ethanol treatment has been shown to enhance astrocyte iNOS expression [36]. In addition, a previous study reported a higher hepatic iNOS mRNA expression in FOXO3-/- mice fed with ethanol [37]. Interestingly, in a study of activated human A172 astrocytoma cells, acute exposure (within 1 day) of low-dose (50 mM) ethanol was shown to enhance iNOS activity recovered from the cytosol, whereas a higher concentration of ethanol (200 mM) decreased iNOS activity [38].

VEGFR2 up-regulation is usually accompanied by eNOS activation in endothelial cells [39]. Nevertheless, a previous study reported that IL-1β induced iNOS expression and significantly up-regulated VEGF mRNA expression and protein synthesis in rat vascular smooth muscle cells [40]. In summary, the response of iNOS or eNOS to alcohol is complex and depends on the cell type, duration of exposure and concentration of alcohol, and disease models.

Interestingly, portal pressure was not significantly influenced by acute or chronic alcohol administration in the non-alcoholic cirrhotic rats in this study. Portal pressure is determined by interactions among hepatic, splanchnic and collateral systems, and both structural and functional components contribute to these three vascular systems. Since the severity of liver fibrosis and hepatic vasoconstrictive response were not significantly influenced by alcohol, two other factors should be taken into consideration, as increased mesenteric angiogenesis and flow can elevate portal inflow and pressure. Nevertheless, collateral vasodilatation helps to relieve stagnant blood flow in the portal system. Taken together, these results indicate that portal pressure is not significantly influenced by alcohol.

Currently, the recommendations regarding alcohol consumption in patients with pre-existing viral hepatitis are inconsistent. For example, abstinence is suggested in all patients with chronic hepatitis C by the American Association for the Study of Liver Diseases (AASLD). However, the European Association for the Study of the Liver (EASL) only suggests stopping harmful alcohol consumption (Audit-C score > 4) [9,10]. On the other hand, the AASLD guidelines suggest abstinence or minimal alcohol ingestion, while this issue is not addressed in the EASL guidelines regarding chronic hepatitis B [11,12]. This may be related to the small number of relevant studies. To date, most previous studies have only focused on the correlation between the amount of alcohol consumption and liver fibrosis. Luca et al. evaluated the effect of a single oral dose of alcohol on hepatic venous pressure gradient (HVPG) in patients with alcohol-induced cirrhosis [41]. In their study, 0.5 g/kg of ethanol, which is equivalent to 30 g of alcohol in a 60 kg man, significantly increased HVPG within hours. They concluded that even a moderate amount of alcohol worsened portal hypertensive syndrome. In the present study, we clearly demonstrated that even low-dose alcohol could exacerbate shunting and splanchnic hyperdynamic circulation without causing significant liver damage. Further investigations are warranted to investigate the effects of alcohol on various kinds of pre-existing liver injuries.

Alcohol intolerance may also occur in liver cirrhosis: Portosystemic collaterals elevate alcohol concentration by bypassing the liver, thus diminishing the amount of alcohol that can be processed. Additionally, previous studies have demonstrated the reduced alcohol metabolic capacity in the liver. In cirrhotic rats, alcohol uptake by the liver was reduced by 30% [42]. However, the influences of long-term alcohol exposure or cirrhosis on alcohol dehydrogenase are inconsistent. A study that recruited alcoholic and non-alcoholic patients with varying severity of liver damage revealed that alcohol dehydrogenase activity decreased in alcoholic cirrhosis rather than in hepatitis. Furthermore, in non-alcoholic cirrhotic patients, alcohol dehydrogenase activity was not affected compared with hepatitis patients [43]. Another study with similar patient characteristics showed that alcohol dehydrogenase activity was reduced in cirrhotic patients, regardless of alcohol consumption [44]. The varied results suggested that the effects of alcohol or cirrhosis on alcohol dehydrogenase are complicated and further study is warranted.

The main limitation of the present study is the lack of a thorough mechanistic survey of the VEGFR2 pathway. Although activation of the VEGFR2 pathway was accompanied by up-regulation of Akt and Erk phosphorylation in the alcohol-administered group in this study, other stimuli and different pathways may also have participated. As a result, whether VEGFR2 is the key pathway involved in low-dose alcohol-induced adverse effects in cirrhosis cannot be ascertained, and further studies are needed to clarify whether targeting the VEGRF2 pathway is a feasible treatment strategy. On the other hand, alcohol impacts males and females differently, with females being more vulnerable to alcohol-related liver disease. This increased vulnerability may be due to differences in alcohol metabolism between the sexes [45]. Furthermore, estrogen enhances Kupffer cell susceptibility to stimulation and results in more severe liver injury [46]. In the present study, a single dose of alcohol induced vasodilatation in portosystemic collateral shunting. Long-term alcohol consumption results in extrahepatic angiogenesis and precipitates splanchnic hyperdynamic circulation. Although the hazardous effects of low-dose alcohol occur in extrahepatic systems, the concentration of alcohol may vary between female and male rats in the present study. Whether low-dose alcohol causes more severe effects in females than in males deserves further investigation.

## Conclusion

In conclusion, low-dose alcohol exacerbated portosystemic collaterals via vasodilatation in non-alcoholic cirrhotic rats in the present study without causing significant liver damage. Furthermore, long-term alcohol use precipitated splanchnic hyperdynamic circulation. Further studies are warranted to evaluate the benefits of avoiding low-dose alcohol consumption in patients with non-alcoholic cirrhosis.

## Supplementary Material

Supplementary Figure S1

## Data Availability

The datasets used and analyzed during the current study are available from the corresponding author on reasonable request.

## Abbreviations

AASLD: American Association for the Study of Liver Diseases
BDL: bile duct ligation
BW: body weight
CI: cardiac index
DW: distilled water
HR: heart rate
HVPG: hepatic venous pressure gradient
MAP: mean arterial pressure
PP: portal pressure
SLS: splenorenal shunt
SMA: superior mesenteric artery
SVR: systemic vascular resistance
VEGF: vascular endothelial growth factor
