# Supplementary material for: Low-dose alcohol exacerbates hyperdynamic circulation and shunting in non-alcoholic cirrhotic rats
Source: Biosci Rep. 2024 Jul 19;44(7):BSR20240354. doi: 10.1042/BSR20240354 (PMC11263042; doi:10.1042/BSR20240354)
Supplement: Supplementary Figure S1 [file BSR-2024-0354_supp.pdf]

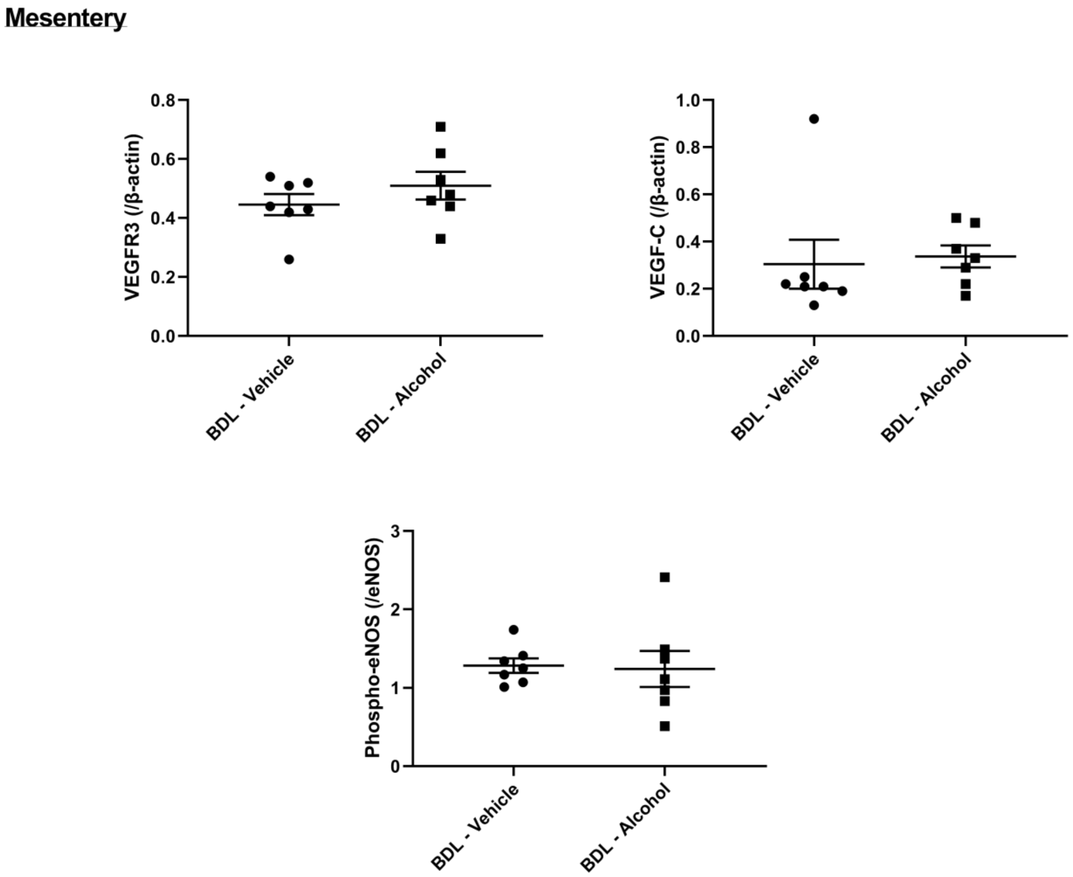

Supplementary figure 1

Protein expressions in mesentery. Chronic alcohol administration did not affect VEGFR3, VEGF-C, or phospho-eNOS expression in mesentery.
